# Comparative evaluation of INNO-LiPA HBV assay, direct DNA sequencing and subtractive PCR-RFLP for genotyping of clinical HBV isolates

**DOI:** 10.1186/1743-422X-7-111

**Published:** 2010-05-30

**Authors:** Maisa M Ali, Fuad Hasan, Suhail Ahmad, Widad Al-Nakib

**Affiliations:** 1 Department of Microbiology, Faculty of Medicine, Health Sciences Center, Kuwait University, Kuwait; 2 Department of Medicine, Faculty of Medicine, Health Sciences Center, Kuwait University, Kuwait; 3 Gastroenterology Unit, Mubarak Al-Kabeer Teaching Hospital, Ministry of Health, Kuwait; 4 Genetics & Genomics Research Unit, Biomedical Research Department, Dasman Diabetes Institute, P.O. Box: 1180 Dasman 15462, Kuwait

## Abstract

Genotypes (A to H) of hepatitis B virus (HBV) influence liver disease progression and response to antiviral therapy in HBV-infected patients. Several methods have been developed for rapid genotyping of HBV strains. However, some of these methods may not be suitable for developing countries. The performance of INNO-LiPA HBV Genotyping assay (LiPA), direct DNA sequencing and subtractive PCR-RFLP of genotype-specific HBV genome regions were evaluated for accurately determining the HBV genotypes by analyzing sera (n = 80) samples from chronic HBV patients. Both, LiPA and DNA sequencing identified 63, 4 and 13 HBV strains as belonging to genotype D, genotype A and mixed genotype A and D, respectively. On the contrary, the PCR-RFLP-based method correctly identified all 4 genotype A but only 56 of 63 genotype D strains. Seven genotype D strains yielded indeterminate results. DNA sequence comparisons showed that a single nucleotide change in the target region generated an additional restriction site for *Nla *IV that compromised the accuracy of this method. Furthermore, all the mixed genotype A and D strains were identified only as genotype A strains. The data show that the PCR-RFLP-based method incorrectly identified some genotype D strains and failed to identify mixed genotype infections while LiPA and DNA sequencing yielded accurate results.

## Findings

**Figure 1:**
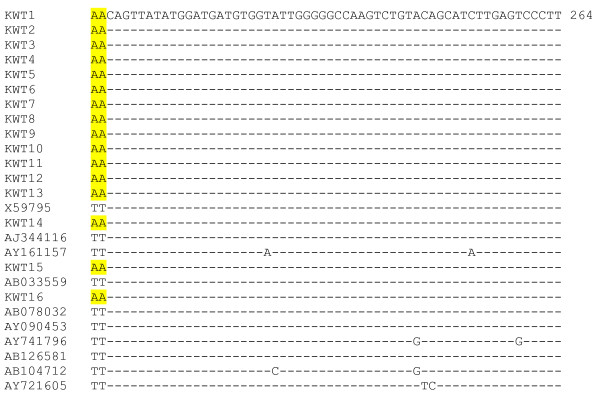
**Alignment of nucleotide sequences of the HBPol gene product of HBV genotype D**. A-to-T nucleotide substitution occurred at nt 204 in all HBV Kuwaiti isolates (KWT1-16). These substitutions represent unique signature sites for local HBV genotype D isolates (shaded). Representative sequences of genotype D are shown (X59795-ITALY, AJ344116-FRANCE, AY161157-INDIA, AB033559-PAPUA, AB078032-JAPAN, AY090453-SWEDEN, AY741796-IRAN, AB126581-RUSSIA, AB104712-EGYPT and AY721605-TURKEY).

**Figure 2:**
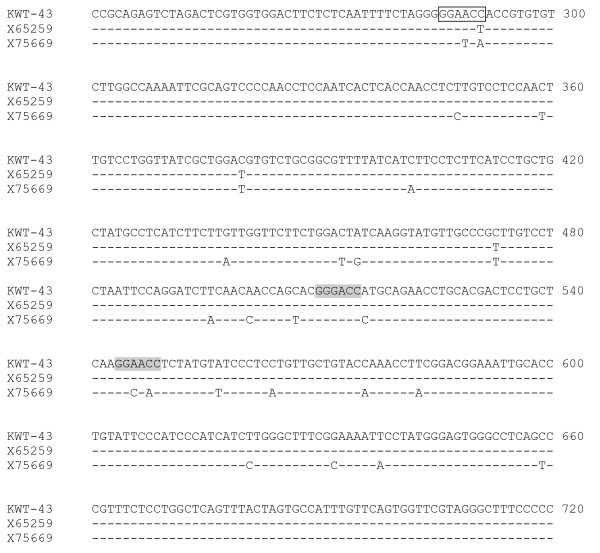
**Alignment of nucleotide sequences of the S-gene product**. Representative sequence of genotypes A (X75669) and D (X65259) are shown at the bottom of the raw. Inherent recognition sites of *Nla *IV in genotype D are shaded. T-to-C nucleotide substitution occurred at nt 291 (box) in KWT-43 (local HBV isolate). These substitutions resulted in generation of a new recognition site of *Nla *IV (motif: GGNNCC).

Eight distinct genotypes (A to H) of hepatitis B virus (HBV) have been identified and their occurrence exhibits distinct preferences for ethnic origin of the patient and/or geographic regions of the world [1,2]. Recent studies have shown that HBV genotypes influence liver disease progression, selection of mutants and response to antiviral therapy in acute and chronic HBV infections [3-5]. Considering the importance of determining the HBV genotype, several methods have been developed for genotyping of HBV strains [6]. A PCR-RFLP-based method that involves successive digestion of amplicon with a battery of restriction enzymes to discriminate the individual genotypes is most suitable for developing countries. However, there is very limited data on its performance in different countries/geographical settings [7,8]. A commercially available reverse hybridization-based line probe assay (INNO LiPA HBV Genotyping assay, LiPA) is easy to perform and is also suitable for detecting mixed genotype infections [9]. Despite these advantages, the test is fairly expensive for resource-poor countries. Direct DNA sequencing of PCR generated amplicons corresponding to genotype-specific regions of HBV genome also yields accurate genotype assignments and is the method of choice for patients infected with recombinant genotypes [9,10]. However, DNA sequencing is still considered as technically demanding, time consuming and costly in most of the developing countries. This study was carried out to evaluate the performance of three (LiPA, direct DNA sequencing and subtractive PCR-RFLP) genotyping methods to determine the method most suitable for routine use in a developing country. The comparative performance of the three methods was tested by using 80 consecutive HBV-DNA positive serum samples obtained from chronic HBV patients. The study was approved by the Committee for the Protection of Human Subjects in Research, Faculty of Medicine, Kuwait University. The HBV DNA was isolated from blood samples by using High Pure Template Preparation Kit (Roche Applied Science, Germany) according to the kit instructions. The final pellet was resuspended in 100 μl of pre-heated elution buffer and 10 μl was used as a template for HBV DNA amplification. The LiPA kits were obtained from Innogenetics (Belgium) and were used according to the instructions supplied with the kit. Briefly, the HBV DNA was amplified by nested PCR, the amplicons were hybridized to genotype-specific probes impregnated on membrane strips and the hybrids were detected with chromogenic substrates. The LiPA results were interpreted as instructed by the kit's manufacturer. The genotype-specific segment of the HBV polymerase gene was amplified and sequenced for DNA sequence-based determination of specific HBV genotypes exactly as described previously [10]. The subtractive PCR-RFLP-based genotyping was performed by semi-nested PCR amplification of a 485 bp fragment which was successively digested with 5 restriction enzymes (in separate tubes) to identify genotype-specific sequences exactly as described earlier [8]. For isolates giving aberrant results, the amplicons were cloned in pGEM-T Easy plasmid as described previously [11]. In each case, the plasmid DNA was isolated from 10 independent clones of *Escherichia coli *by a modified alkaline lysis procedure [12] and sequenced by using quick start cycle DNA sequencing kit (Beckman-Coulter). The DNA sequencing was performed as described in detail previously [13,14]. The LiPA identified 63 and four HBV strains as belonging to genotype D and genotype A, respectively. Thirteen HBV strains yielded amplicons that hybridized to probe primers specific for both, genotype A and genotype D, and were thus identified as mixed genotype A and D strains. The DNA sequencing data of genotype-specific region of HBV polymerase gene correlated completely with the genotype assignment obtained with LiPA for all the 80 strains. The results are consistent with earlier reports showing the ability of the LiPA to detect mixed genotype infections [9,15,16]. The comparative analyses of DNA sequence data for all genotype D strains in Kuwait (data from 10 selected isolates are shown in Figure 1) identified a unique signature of AA dinucleotide at position 204 and 205 in the HBV polymerase gene. This signature sequence is not only absent in representative genotype D strains isolated in Italy, France, Russian Federation, Sweden, India, Japan and Papua New Guinea [GenBank: X59795, AJ344116, AB126581, AY090453, AY161157, AB078032 and AB033559; respectively] but also from several Middle Eastern countries such as Iran, Egypt and Turkey [17-19] (Figure 1). Since the genotype-specific region of the HBV polymerase gene overlaps with the surface antigen of the HBV (HBsAg), the sequencing data can also be used to deduce the "a" determinant (amino acids 124-147) of the S-gene specifying all the subtypes [20]. Based on these analyses, three genotype D strains displayed unusual amino acid substitutions. Two isolates shared the amino acid pattern Arg^122^, Pro^127 ^and Ser^140 ^specifying *ayw *subtype with unique amino acid substitution involving residue 134 (Ile^134 ^or Asn^134^). The remaining strain contained unique amino acid substitution at codon 127 (Asn^127^). Furthermore, all the mixed genotype A and D strains were untypeable, as expected. The subtractive PCR-RFLP-based assay showed that none of the strains belonged to genotype C, B, E or F. Following *Nla *IV digestion, the 220 bp fragment characteristic for genotype A was observed with 17 HBV strains. Four of these strains were identified as belonging to genotype A by LiPA or DNA sequencing while the remaining 13 strains were identified as mixed genotype A and D strains by the other two genotyping methods. The data showed that the PCR-RFLP-based method detected Genotype A status of all HBV strains whether due to monoinfection with Genotype A (n = 4) or due to mixed Genotype A and D infection (n = 13). Only 56 of the remaining 63 strains yielded a 186 bp fragment characteristic for genotype D following restriction digestion with *Nla *IV while the remaining seven strains yielded atypical RFLP patterns (a ~220 bp fragment was present in addition to the expected 186 bp fragment) (data not shown). The 485 bp fragment from all the seven isolates was, therefore, cloned in pGEM-T Easy plasmid and 10 independent recombinant plasmids for each HBV strain were sequenced. The DNA sequencing data showed that all the seven strains contained a T to C nucleotide substitution at nucleotide position 291 that generated a new recognition site of *Nla *IV within the larger (265 bp) fragment that is ordinarily released following *Nla *IV digestion of 485 bp amplicon from genotype D strains (Figure 2). Thus, the presence of an additional restriction site for *Nla *IV, resulting in further digestion of 265 bp fragment into 41 bp and 224 bp fragments, yielded aberrant results by PCR-RFLP-based method. These findings are similar to an earlier report from Uzbekistan. The PCR-RFLP-based assay yielded aberrant results for five of 54 HBV strains [7]. Similar to the results reported in this study, their DNA sequencing data for two of the five strains also confirmed the presence of T to C substitution at the same site that generated an additional restriction site for *Nla *IV [7]. In conclusion, the results of this study showed that subtractive PCR-RFLP-based genotyping of HBV strains is simple to perform and accurately detected genotype assignment of a substantial number (60 of 80) of HBV strains. Additionally, the PCR-RFLP-based method also detected the Genotype A in all 13 mixed Genotype A and D strains of HBV. On the other hand, despite being costly or technically demanding, LiPA and direct DNA sequencing yielded accurate identification of all genotype A and genotype D and mixed genotype A and D strains of HBV. The DNA sequencing data reported in this study have been submitted to [GenBank under accession numbers: AM279420 to AM279439].

## Competing interests

The authors declare that they have no competing interests.

## Authors' contributions

MMA, FH, SA and WAN designed the study and FH collected the specimens. MMA carried out the DNA Extraction, INNO-LiPA genotyping assay, Direct DNA Sequencing, PCR-RFLP studies, sequence alignments and analysis. SA participated in the sequence alignment analysis. All authors contributed in manuscript writing, read and approved the final manuscript.
